# Research challenges in digital education

**DOI:** 10.1007/s40037-014-0139-7

**Published:** 2014-08-21

**Authors:** Geoff Norman

**Affiliations:** 1 Canada Research Chair in Cognitive Dimensions of Clinical Expertise, McMaster University, Hamilton, ON Canada; 2 Program for Educational Research and Development, McMaster University, Room 3519, MDCL 1200 Main St West, Hamilton, ON L8N 4K1 Canada; 3 Department of Clinical Epidemiology and Biostatistics, McMaster University, 1280 Main St. W., Hamilton, ON Canada; 4 Department of Psychology, McMaster University, Hamilton, ON Canada

**Keywords:** E-learning, Simulation, Instructional design, Fidelity

## Abstract

Simulation and other forms of digital learning will occupy a place of increasing prominence in medical education in the future. However, to maximally use the potential of these media, we must go beyond a research agenda dictated by a ‘Does it work?’ question to one driven by careful analysis of the nature of the task to be learned and its relation to the characteristics of the technology. Secondly, we must change the focus from the characteristics of individual devices to a broader approach to design of a digital curriculum based on current understanding of the nature of human learning.

I have more than a passing interest in the papers presented in this issue of Perspectives, for two reasons. First, I am more and more convinced that educational technology—online learning, virtual patients, simulations—is an essential element in avoiding the demise of quality medical education in the face of irrevocable changes in the health care system. I have written about this issue in a previous paper in Perspectives [1]. Second, and more immediately, I was just discharged from hospital today where I underwent an emergency laparoscopic cholecystectomy. So the articles on simulation in learning laparoscopic skills are of more than passing interest. Indeed, in the many hours spent in emergency department waiting rooms and hospital beds, I have had more opportunity to see the products of our educational efforts at work than at any time in the past three decades.

To frame the discourse, I have two take-home messages:

1. Digital technology can, and must, have an essential role in curriculum in health sciences in the future if our educational systems are to remain economically viable.
2. However, much of our research is asking the wrong question. And unless we address the critical questions, the opportunity will be lost.

Them’s fightin’ words, I know. So it is time to defend myself.

Let me begin with some observations from my recent hospitalization. In order to pass the many idle hours, I tried to observe the students and professionals with a view to understanding the kind of expertise they had, and what educational events would be required to attain that expertise. One expertise that you see far too often when you’re in hospital is finding a vein. Whether it’s to start a drip or draw blood for tests, by the time you leave you have renewed sympathy for pincushions. What does it take to acquire this skill? It seemed to me based on my recent experience that, counter to my expectations, much of the expertise was perceptual—visual perception to find possible veins followed by an acute sense of touch to feel the right degree of elasticity. One nurse, in particular, scanned my arms for likely candidates like a cat stalking a field of sparrows. She would then gently touch the candidate vein to see if it had the right rebound. When she finally did insert the needle, she never missed.

When the junior medical student asked if he could examine my chest, I responded, ‘Why?’ He says he never misses the opportunity to listen to a heart, as he knows he will take a very long time until he has heard enough heart sounds to become competent at auscultation. I asked, ‘How often do you hear an abnormal sound?’ He replied, ‘About once a week.’ ‘And can you tell what you’re listening to?’ ‘Not often.’ How could he? He gets no feedback.

Both of these skills are favourites for simulation. For IV insertion, you can run the gamut from a pig’s foot, to a static plastic simulator, to a virtual reality simulator, to SimMan and its variants; the cost ranges over many orders of magnitude. But NONE of the simulators addresses the perceptual skill that the nurse displayed in scanning for veins. When I asked her how many a day she does, it was 10–20. And when I asked her how long it took her to get good at it, it was six months to a year. That is somewhere from 1,000 to 4,000 attempts. It is clear that she could not achieve anything like this degree of mastery by poking SimMan’s arm 4,000 times.

Similarly for heart sounds. A student has a vast choice of simulations, from free heart sounds downloaded from the Web, to Harvey, at $50,000 (about 35,000 Euro). While the medical student’s self-directed experience was inadequate, since he had no way to get feedback, he did recognize that to achieve mastery he was going to have to listen to a great many heart sounds. Harvey has 29—one of each condition.

And of course laparoscopy has been the subject of many simulation approaches, two of which are reported in this issue. It is more difficult for me to comment on the expertise involved in this skill, since I slept through the whole thing. But as I listened to the surgeon’s explanations before the procedure, it was evident that his expertise also amounted to direct experience with many, many variations on cholecystitis.

It seems to me that research and development in simulation has not been guided by any careful analysis of the critical elements of the task. Instead, we have adopted the maxim that more (fidelity) is better, and accepted uncritically that somehow if a simulator looks realistic, it will get the job done better than one that does not. Moreover, far too much of the research on simulation to date has focused on whether or not it shows gains compared with some kind of placebo instruction. As Ellaway [2] says in this issue:

We clearly need to move beyond the ‘works’/’not works’ discourse of much of the existing scholarship around virtual patients, and for that matter, around educational technologies as a whole.

Cook et al. [3] distinguish three kinds of research in this area: description, justification and clarification. The critical distinction is between the last two: justification asks, ‘did it work?’; clarification asks ‘why or how did it work?’ In their review, only 12 % of studies fell into the clarification domain.

 I suggest we must go beyond this distinction in two critical ways. First, we should begin with a task analysis, and identify the critical elements of the task. As I reflect on the areas where simulation is applied, it seems to me that for many of the tasks for which we create simulators, there are two critical and separable domains: perceptual and motor. In auscultation, I suspect it takes very little instruction to learn how to hold a stethoscope, how to position the patient, and so on, and many, many more hours to distinguish the various ways different heart sounds can present. Until this week, I might have thought that IV insertion represented the opposite balance, with the emphasis on motor skill and the deft insertion of the needle into the vein. But what I saw was that the critical skill was to identify by inspection and touch the best site to insert the needle. Of course, one must also consider the level of the learner. In an area like ophthalmology, there is no point in trying to identify the subtleties of macular degeneration or retinal detachment if you cannot see the retina. Some mastery of motor skill, which in the case of ophthalmological examination is not easy, is necessary before one can capitalize on the variation in perceptual presentations.

Treating fidelity as a unidimensional construct itself is too simplistic. What we need to do is decompose the subtasks in a skill domain, then build a simulator curriculum around them, taking the specific characteristics of each subclass into consideration. We must design the curriculum around the prerequisite skills, not the available simulators. In the case of a skill like auscultation or IV insertion, we have to decouple the learning of the perceptual skills from the motor skills. To learn how to hold a stethoscope or to insert a needle will require one kind of simulator which accurately simulates the anatomical structures both visually and haptically; to learn how to recognize the different heart sounds may require an entirely different simulator that contains sets of confusable heart sounds on an iPod or flash drive. As to learning how to recognize the right kind of vein to insert an IV, I venture that adequate simulation of this perceptual skill is beyond the capacity of any of our technologies, while the motor skill of needle insertion can be simulated with many devices ranging in price from hundreds to tens of thousands of dollars [4].

Theory of simulation is beginning to move from description and justification to a detailed consideration of the simulation as a component of a system of instruction. Ellaway’s [2] paper in this issue exemplifies this perspective, as does a recent paper by Grierson [5]. However I do not see that this progression has arisen in empirical research in the area, with a few notable exceptions [4]. We need to go further, as Ellaway [2] suggests. There is, I believe, ample evidence to show that the gains from greater authenticity are not commensurate with the cost; whatever you can get with a hi-fi simulator, you can get most of it with a lo-fi simulator as well [6, 7]. Of course, the same concern can be raised about the hyper-authenticity embodied in the ‘Augmented Reality’ simulators described by Kamphuis et al. [8]. It may well be advantageous to use the simulator to reveal underlying structures and mechanisms. Baghdady et al. [9]. have shown a value of basic science concepts in interpreting even such perceptual stimuli as dental X-rays, so it is possible that the provision of information about underlying structures may increase performance. But this cannot be assumed; the extra information may increase cognitive load and decrease performance.

Which brings me back to the skill of the laparoscopic surgeon or the IV nurse. If my analysis is correct, then a focus on the motor skills by practice with the **same** stimulus—the one inflamed appendix in the VR lap simulator or the one plastic vein in the static IV manikin—is inadequate. Dealing with variability is the essence of expertise [10]. If we are to maximally use the potential of simulation, the simulator must be designed to capture this variability. We should present students with not one mitral stenosis on a heart sound simulator, or one acute cholecystitis on a virtual patient, but with dozens and dozens, systematically varying and interleaved with examples of other conditions—mixed practice, which has been shown to consistently provide large benefits [11]. This is clearly within the capability of the technology, yet is rarely exploited. It is this suggestion which goes beyond the simple equating of a simulator with a skill and brings in the multiple dimensions described by Ellaway [2].

Goris [13] and colleagues describe an innovative game that is carefully designed to facilitate learning laparoscopic skills in an engaging format. He states that ‘a number of controlled experiments have shown that video games can be used to increase basic laparoscopic skills in novices in the short- and middle-long term’. This leaves unanswered the question: ‘As compared to what?’ As I review the manuscript, I feel a certain ambivalence. On the one hand, I can appreciate that the game they have devised is engaging and clever, and the careful mapping of game skills onto laparoscopic skills is admirable. It may be that an engaging game such as this may enhance motivation (and we know very little about the role of motivation in learning) as well as facilitating skill development. On the other hand, the game is clearly a ‘low fidelity’ simulation of laparoscopy, and the opportunity to do the manipulations around realistic anatomical structures, and thereby acquire functional anatomical knowledge, has been lost. The relative advantage of increased motivation and increased authenticity can only be resolved through further careful study.

I have not as yet commented on the other domain of digital education represented in this issue; the ‘blended learning’ approaches. The same ‘as compared to what?’ question arises here. As both Dankbaar et al. [14] and De Jong et al. [15]. indicate, there is substantial evidence that students learn as well from an online resources as from face-to-face instruction. This is consistent with a number of systematic reviews which have shown that students almost always learn as well from A as from B. As Cook [12] says, ‘If you teach they will learn.’ We can take as one of the few universal truths in education that any format is more or less equivalent to any other. This leads us in two opposite directions. On the one hand, we could say, ‘Well, we can be confident that students will learn regardless of what we do to them. So let’s design a curriculum for efficient use of faculty time, or distance learning, or universal access, or whatever, and not worry about issues of learning.’ Dankbaar’s study in this issue [14] is an example, where learning outcomes were similar, but the cost of delivery was reduced by 30 %. Alternatively, we could take Cook’s [3] taxonomy to heart and explore deeper just what is the same and what is different about learning from a computer. Surely some things can be learned in various formats. Memorizing a Shakespearean soliloquy likely presents the same difficulty whether it is on a laptop screen or in a book. Conversely, there may well be some areas where the specific affordances of computers can facilitate learning, although the work of Mayer [16] and Van Merrienboer [17] reminds us that common sense may lead us hopelessly astray in this respect. Dynamic simulations, background music, popups, all make things much worse, as they increase extraneous load. In any case, the nature of these questions leads us to clarification research, which holds promise to lead to greater ultimate efficiencies.

Simulation, virtual patients and e-learning are here to stay, and we can be grateful that they are. The challenge is to accept that there is no ‘one size fits all simulator,’ that frequently ‘less is more’ or at least isn’t much less, and to go beyond a preoccupation with proving that a particular simulator works in two opposite directions—down to the basic elements to understand better what are the active and critical elements in matching a technology to a learning situation, and up to give serious consideration to building a curriculum that incorporates multiple kinds of simulations based on an overall design.
